# Effects of *Septin-14* Gene Deletion on Adult Cognitive/Emotional Behavior

**DOI:** 10.3389/fnmol.2022.880858

**Published:** 2022-04-29

**Authors:** Kuan-Ru Chen, Han-Yu Wang, Yi-Han Liao, Li-Han Sun, Yu-Han Huang, Lung Yu, Pao-Lin Kuo

**Affiliations:** ^1^Department of Obstetrics and Gynecology, National Cheng Kung University Hospital, National Cheng Kung University, Tainan, Taiwan; ^2^Department of Obstetrics and Gynecology, National Cheng Kung University College of Medicine, Tainan, Taiwan; ^3^Institute of Basic Medical Sciences, National Cheng Kung University College of Medicine, Tainan, Taiwan; ^4^Department of Physiology, National Cheng Kung University College of Medicine, Tainan, Taiwan

**Keywords:** sex dimorphism, aversive, observational learning, anxiety, neurogenesis

## Abstract

While various septin GTPases have been reported for their physiological functions, their roles in orchestrating complex cognitive/emotional functions in adult mammals remained scarcely explored. A comprehensive behavioral test battery was administered to two sexes of 12-week-old *Septin-14* (SEPT14) knockout (KO) and wild-type (WT) mice. The sexually dimorphic effects of brain SEPT14 KO on inhibitory avoidance (IA) and hippocampal mGluR5 expression were noticed with greater IA latency and elevated mGluR5 level exclusively in male KO mice. Moreover, SEPT14 KO appeared to be associated with stress-provoked anxiety increase in a stress-related navigation task regardless of animals’ sexes. While male and female WT mice demonstrated comparable cell proliferation in the dorsal and ventral hippocampal dentate gyrus (DG), both sexes of SEPT14 KO mice had increased cell proliferation in the ventral DG. Finally, male and female SEPT14 KO mice displayed dampened observational fear conditioning magnitude and learning-provoked corticosterone secretion as compared to their same-sex WT mice. These results, taken together, prompt us to conclude that male, but not female, mice lacking the *Septin-14* gene may exhibit increased aversive emotion-related learning and dorsal/ventral hippocampal mGluR5 expressions. Moreover, deletion of SEPT14 may be associated with elevated ventral hippocampal DG cell proliferation and stress-provoked anxiety-like behavior, while dampening vicarious fear conditioning magnitudes.

## Introduction

Transcription and translation of *Septin* genes are especially eminent in the embryo and neonatal brain, suggesting that *Septin* genes may play a critical role in guiding, scaffolding, and trimming the developing neuronal circuits and modules in the early-life brain (Kumar et al., 1992; Ito et al., 2020). For instance, deletion of the *Septin-14* gene results in deficiency of its protein and disrupted cortical neuronal migration (Shinoda et al., 2010). Moreover, deletion of several subfamilies of *Septin* genes has been proven to affect neuronal dendrite/axon growth/branching, spine morphogenesis, and synaptic formation (Tada et al., 2007; Xie et al., 2007; Li et al., 2009; Cho et al., 2011; Tsang et al., 2011; Hu et al., 2012). Likewise, a growing body of evidence supports the notion that Septin proteins are paramount in their importance in regulating neurophysiological function in the mature brain (Maimaitiyiming et al., 2013; Ito et al., 2020). Several Septin proteins seem to be involved in vesicle transport, neurotransmitter release, and neurogenesis (Bai et al., 2016; Karasmanis et al., 2018; Li et al., 2019). Septin-14 protein is documented to play a critical role in regulating dopamine release and reuptake in the nigrostriatal dopaminergic synapse (Ihara et al., 2007). Septin proteins have been reported to associate with glutamate reuptake and availability in glutamatergic peri-synapses (Ageta-Ishihara et al., 2015). A single *Septin* gene deletion does not necessarily affect mammals’ fatality or fertility (Shafipour et al., 2014). The necessity of a specific *Septin* gene and protein in orchestrating cognitive/emotional function and behavior in adulthood remains mostly unknown (Maimaitiyiming et al., 2013; Ageta-Ishihara et al., 2018; Ageta-Ishihara and Kinoshita, 2021).

The *Septin-14* gene and protein have been reported to be associated with the development of various neuropathologies (Rozenkrantz et al., 2016; Bijak et al., 2019). These results imply that the *Septin-14* gene and its product protein expression may also affect the intactness of cognitive/emotional function. In theory, *Septin-14* deletion-primed brain neuropathology and impeded function may be manifest and reflected, to some extent, in adult animals’ aberrant behavior. Moreover, a family of Septin proteins has been linked to developmental brain pathologies of schizophrenia (Wang et al., 2018). Likewise, *Septin* gene deficiency may cause active affiliative social interaction deficits in an animal model (Harper et al., 2012). Social interaction abnormality is a key symptomatic component of schizophrenia and an autistic spectrum disorder. To tangibly assess the permissive roles of *Septin-14* gene play in processing multi-circuit-based cognitive/emotional and social interaction function, adult wild-type (WT) and *Septin-14* knockout (SEPT14 KO) mice of two sexes were used in this study. A custom-made behavioral task battery was used [including object location, sociability/social novelty preference, inhibitory avoidance (IA), observational fear conditioning, tail suspension, and stress-related free navigation tasks] to assess the impact of SEPT14 KO on these complex cognitive/emotional/social functions (Tzeng et al., 2018; Liao et al., 2021a,b).

Neural stem cell proliferation in the adult hippocampal dentate gyrus (DG) has been implicated in a variety of brain cognitive/emotional functions, ranging from newly formed memory consolidation (Deng et al., 2010; Anacker and Hen, 2017; Miller and Sahay, 2019), stress-provoked emotional responses (Scholpa et al., 2016; Anacker et al., 2018) to social behavior (Snyder and Drew, 2020). To date, few Septin proteins have been identified as key biological substrates participating in the differentiation of proliferated cells into neuroblasts at the embryonic stage (Falk et al., 2019; Ferreira and Garcez, 2019). However, it was unknown whether mature brain Septin-14 may enduringly affect neural stem cell proliferation in hippocampal DG, which is a primary source of adult-born neurons in the mature brain. Moreover, pioneering investigators have posited that activation of ionotropic and metabotropic glutamate receptors confer neural stem cells’ microenvironment to bias toward active proliferation in hippocampal DG (Di Giorgi-Gerevini et al., 2005; Nochi et al., 2012; Thakker-Varia et al., 2014; Shtaya et al., 2018). Furthermore, glutamate GluA1, GluA2, and mGluR5 receptors have been documented to play critical roles in the consolidation of conditionings, spatial learning and memory, exploratory behavior, and social interactive behavior (Jia et al., 2001; Schiavi et al., 2022). Thus, mitotic (Ki-67) marker-labeling and Western immunoblotting methods were exploited to reveal the modulating effects of SEPT14 KO on neural stem cell mitosis in dorsal and ventral DG and local glutamate GluA1, GluA2, and mGluR5 receptor expressions in adult mice.

## Materials and Methods

### Animals

Using the CRISPR-Cas9 system, *Septin-14* floxed mice were generated from the Transgenic Mouse Models Core of National Taiwan University, while the *Sox2-Cre* mice from the Jackson Laboratory (Hayashi et al., 2002; Chen et al., 2019). Mice with germline deletion of *Septin-14* intron 3–5 [i.e., SEPT14 knockout (KO) mice] were obtained by crossing the *Septin-14* floxed and *Sox2-Cre* mice (Supplementary Figure 1). Since the primers, namely, 5VF1: 5′-TTCCAA TTGATTTTGTCTGTGTCA-3′, 5VR1: 5′-AAAATGCCATGATAAACC CCAGG-3′, 3VR1: 5′-CGGTAGGAGACATGATAGCCAAG-3′, were used to identify the WT and SEPT14 KO alleles, the base pairs of recognized WT and KO were 746 and 579, respectively. This study was performed in accordance with the National Institutes of Health Guide for the Care and Use of Laboratory Animals revised in 2011. All procedures were approved by the Local Animal Care Committee at National Cheng Kung University College of Medicine (NCKUCM No. 107172). After their weaning (Day 21 postpartum), male and female WT and SEPT14 KO mice were group-housed in plastic cages (2–5 per cage) in a temperature- and humidity-controlled colony room on a 12-h light/dark cycle with lights on at 07:00. Mice had access to food (Purina Mouse Chow, Richmond, IN, United States) and tap water *ad libitum* throughout the experiments. Since object location and sociability and social novelty preference tasks were minor in their stress-provoking nature, a total of 45 (20 WT and 23 SEPT14 KO) mice received object location, followed by sociability and social novelty preference task, at a 2-h interval at the same day. These mice, then, received inhibitory training and testing for the following 2 days. Nonetheless, it was of importance to note that WT and KO mice were not repeatedly used for the remaining behavioral tasks or bioassays.

### Real-Time PCR

Total RNA isolated from brain tissues was extracted using Direct-zol RNA MiniPrep kit (Zymo Research), and cDNA was synthesized using PrimeScript™ RT reagent Kit (TaKaRa). cDNA was amplified by PCR using specific primers (GAPDH sense 5′-AAGGTCATCCCAGAGCTGAA-3′; GAPDH antisense 5′-TGCCTGCTTCACCACCTTCT-3′; SEPT14 sense 5′-ATGGCGGAAAAACCAACTAACA-3′; SEPT14 antisense 5′-GGAGGTAGGCTTCAAACTGA-3′).

### *In situ* Hybridization (BaseScope)

*Sept14* BaseScope probe targeting 297–446 of NM_028826.2 was designed by Advanced Cell Diagnostics. *Sept14* mRNA in the paraformaldehyde-fixed brain section was detected by BaseScope according to the manufacturer’s instructions (BaseScope™ Detection Reagent Kit-RED User Manual, Advanced Cell Diagnostics). The images were acquired using a VENTANA DP 200 slide scanner (Roche).

### Sociability and Social Novelty Preference

To assess mice’ social recognition and novelty, a custom-made three-compartment chamber (42 cm × 21 cm × 21 cm) was used (Liao et al., 2018). In brief, the test consisted of three sessions, including habituation, sociability, and social novelty preference sessions. In the 10-min habituation session, female and male WT and SEPT14 KO mice were individually subjected to the center compartment (12 cm × 21 cm × 21 cm) starting unrestrained navigation in the chamber [consisting of a center and two side compartments (15 cm × 21 cm × 21 cm)]. In the sociability session, an unfamiliar, same-sex C57BL/6 mouse (stranger 1) was placed in one randomly chosen side compartment enclosed in a wire cage (7 cm × 7 cm × 10 cm) that allowed for air exchange and likely olfactory, auditory, and visual interactions. An empty, but otherwise identical, wire cage was placed in the opposite side compartment. Mice were allowed to explore the chamber for social preference for a 10-min period. After the conclusion of the sociability session, the mice were immediately returned to the center compartment, and the doors to the two side compartments were blocked. With stranger 1 retained in its original side compartment, another unfamiliar, same-sex mouse (stranger 2) was placed in the previously empty wire cage in the opposite side compartment. Starting the social novelty preference session, both doors to the side compartments were unblocked and mice were allowed to explore the entire chamber for their novelty preference for another 10-min period. Mouse position and motion were tracked using a top-mounted charge-coupled device connected to a computer, which was recorded and analyzed using Logitech Webcam Software. The exploration time spent (in second) in close proximity (less than 1 cm) of the mouse nose directing to the empty, stranger 1 and 2’s wire cages were recorded. Likewise, individual mouse stranger 1 and stranger 2 preference ratios (i.e., time spent with stranger 1/total time spent with empty and stranger 1 and time spent with stranger 2/total time spent with stranger 1 and 2) were obtained.

### Object Location Task

The object location task consisted of 30-min habituation, 10-min training, and a 10-min test session (Tzeng et al., 2016). Regardless of sex and SEPT14 KO, mice first received the habituation session individually freely navigating in an empty Plexiglas box (46 cm × 26 cm × 21 cm) with black walls and a bright yellow floor in a dimly lit (<40 lux) laboratory for 30 min. Two teacups, being placed upside down, were then placed in the opposite corners sharing the same wall, with the cup border 6.8 cm away from the walls of the box in the same dimly lit laboratory for the 10-min training session. In this training session, mice were allowed to freely explore in the box and the time mice spent exploring two cups was recorded. Mice were, then, transported to holding cages for an approximate 5-min waiting period following the training session. Starting the test session, the mice were returned to the box with one cup moved to a diagonally opposite corner, with a cup border also 6.8 cm away from two adjacent walls. The recognition percentage, which was the ratio of the time spent exploring the cup in the new corner over the total time spent exploring both cups, was used to determine the object location memory. It was of importance to note that boxes and cups were thoroughly cleaned between adjacent sessions to stop the likely build-up of olfactory cues.

### Open Field Task Under Bright Light Condition

The stress-related navigation was gaged in a custom-made Plexiglas chamber with a white floor and dark walls (41 cm × 41 cm × 30 cm). A 13-W incandescent light was used 60 cm above the chamber to provide illumination at the bottom center (900 lux) throughout the test. Their time spent in the defined central zone (20 cm × 20 cm) and the remaining zone were recorded using the Noldus Ethovision XT video tracking system (Noldus Information Technology, Beijing, China). In addition, mice running speed and navigation distance were also obtained using such system-harboring software.

### Observational Fear Conditioning Task

A custom-made chamber with two compartments was used, consisting of a footshock-delivering compartment and an adjoining insulated observational compartment (Liao et al., 2021b). Three same-sex C57BL/6 mice served as demonstrators, receiving a daily footshock protocol (pseudorandomly scheduled 15 footshocks in total, 0.5 mA in intensity, lasting 2 s each, delivered in 30 min) in the shock-delivering compartment (a 24-cm long, trough-shaped metal alley) and a cotton tip impregnated with vanilla extract was taped onto the compartment ceiling throughout the footshock protocol. Individual female and male WT and SEPT14 KO mice were the observer mice being subjected to the adjoining observational compartment (a 16-cm long, trough-shaped metal alley) throughout the demonstrators’ daily footshock protocol. The shock-delivering compartment was separated from its adjoining observational compartment by a transparent and perforated Plexiglas divider, allowing observer mice’ visual, auditory and olfactory cue reception (Liao et al., 2021b). The aforementioned daily footshock sessions were administered for 3 consecutive days. Approximately 24 h following the conclusion of the training session, freezing baseline and vanilla odor-induced freezing responses (FRs) were assessed in a novel test environment to reveal the magnitudes of novelty- and observational fear conditioning-induced FRs, respectively. A 10-min freezing baseline was first obtained in a transparent test chamber (28.5 cm × 17.5 cm × 11.5 cm) to reveal observers’ differences in their novelty-related FR. These observer mice were then individually subjected to a 30-min vanilla odor-induced FR test in another chamber (28.5 cm × 17.5 cm × 11.5 cm), wherein a cotton tip impregnated with vanilla extract was taped on the ceiling (Liao et al., 2021b). An FR was defined as a complete cessation of all movement except for respiration. FRs were sampled every 10 s throughout the 30-min videotaping and were analyzed by an experienced rater blind to the groups. Importantly, the mouse serum corticosterone (CORT) assay was done following the first observational fear conditioning as previously mentioned (Liao et al., 2021b).

### Inhibitory Avoidance Task

The custom-made step-through IA apparatus consisted of a trough-shaped metal alley (bottom width = 1.7 cm) divided by a sliding door into an illuminated safe compartment (17 cm in length with a transparent lid) and a dark shock delivery compartment (24 cm in length with an opaque dark brown lid) (Tzeng et al., 2012). A shock generator (SMSCK, Kinder Scientific Com LLC, Poway, CA, United States) was connected to the alley floor of the dark compartment. In PA training, mice were individually placed at the far end of the illuminated compartment facing away from the door. As the mouse turned around and entered the dark compartment, the door was manually closed and a 0.5-mA footshock was given for 3 s after a 3-s delay. The mouse was left in the dark compartment for another 10 s, then removed from the apparatus and returned to the home cage. The latency of dark compartment entry in the training was used as a baseline for each mouse. Approximately 24 h later, mice were individually placed into the illuminated compartment, and the latency to step into (with all four paws) the dark compartment was recorded as the measure of retention time indexing IA memory magnitude.

### Tail Suspension Task

Mice were suspended upside down by the tip of their tail on a custom-made horizontal rod approximately 45 cm away from the bench (Chuang et al., 2011). When mice were held in such an upside-down position, the immobile posture was used to index their giving-up responses in the face of inescapable stress. Accordingly, the duration of immobility (remained to be immobile for at least 1 s) in a 6-min test was videotaped and analyzed by a rater blind to the mouse groups.

### Immunohistochemical Staining Protocol and Quantification for Newly Proliferated Cells

To label newly mitotic cells in hippocampal DG, Ki67-staining methods were used in both sexes of WT and SEPT14 KO mice (Wan et al., 2019). In brief, mice were deeply anesthetized with sodium pentobarbital and transcardially perfused with ice-cold 0.1 M phosphate-buffered saline (PBS, pH adjusted to 7.4), followed by 4% paraformaldehyde in ice-cold 0.1 M PBS. Brains were removed and postfixed in a 4% paraformaldehyde solution overnight at 4°C and subsequently cryoprotected in a 30% sucrose solution for 48 h at 4°C. Coronal sections at 20 μm in thickness were made using a microtome (Shandon Cryotome E, Runcorn, Cheshire, United Kingdom). The paraformaldehyde-fixed brain section was stained with rabbit anti-mouse Ki67 (1:200, Chemicon, Temecula, CA, United States) antibody for newly mitotic cells. The immunohistochemical staining was made using the vectastain ABC system (Vector Laboratories, Burlingame, CA, United States). Thus, the brain sections were further incubated with Rhodamine (TRITC) conjugated goat anti-rabbit (1:200, Chemicon, Temecula, CA, United States) secondary antibody and imaged with a Zeiss fluorescent microscope. Staining was quantified in the dorsal (bregma: −1.06 to −2.06 mm) and ventral (bregma: −3.08 to −3.80 mm) hippocampal DG (Franklin and Paxinos, 1997). It was important to note that a Ki67-labeled spot was regarded as a newly proliferated cell by using the standard that its location was aligned with the internal border of the granule cell layer. Ki67-positive cells in every seventh section were added and then divided by the slide selection ratio to obtain the total number of labeled cells for the defined range of the dorsal and ventral hippocampal DG.

### Western Immunoblotting

Mice were deeply euthanized by intraperitoneal chloral hydrate (40 mg/kg, Sigma, St. Louis, MO, United States), followed by sodium pentobarbital (80 mg/kg, SCI Pharmtech, Inc., Taoyuan, Taiwan). Mice, then, underwent rapid decapitation, and their brains were removed and placed on the dorsal surface of a glass dish sitting on crushed ice. Coronal brain slices (at 1 mm in thickness) were made using a mouse brain slicer matrix (Zivic Instruments, Pittsburgh, PA, United States). Brain tissues containing the dorsal and ventral hippocampus were dissected as previously reported (Deng et al., 2010). These tissue samples were homogenized in an ice-cold lysis buffer containing a protease inhibitor cocktail (Roche, Basel, Switzerland). Homogenates were centrifuged at 12,000 × *g* for 10 min at 4°C, and the protein concentrations of supernatants were determined by the Bradford method (Bio-Rad Protein Assay, BioRad Laboratories, Hercules, CA, United States) using bovine serum albumin as standards. Samples were heated for 5 min at 95°C in a 2 × sample buffer. The electrophoretically separated proteins were transferred to PVDF membranes (Perkin-Elmer™ Life Sciences, Boston, MA, United States) and blocked with 5% non-fat milk in phosphate saline-Tween-20. The membranes were then incubated overnight at 4°C with the primary antibodies, including rabbit-anti-mouse GluA1 (1:1,000, Merck Millipore) and GluA2 (1:2,000, Merck Millipore), mGluR5 (1:10,000, Abcam), and anti-β actin (1:10,000) antibody (Merck Millipore; Middlesex County, MA, United States). Following incubation with goat anti-rabbit secondary antibody (1:5,000) from Jackson ImmunoResearch Laboratories (West Grove, PA, United States), the blots were developed with the ECLTM Western blot detection kit (Perkin-Elmer™ Life Sciences, Boston, MA, United States). Band density of the protein of interest was quantified using ImageJ image analysis software and expressed as the relative intensity of the respective protein of interest against β actin.

### Statistical Analysis

Using male and female WT mice’ training baseline and test latency, a paired Student’s *t*-test was employed first, serving as *a priori* test, to assess the validity of IA training. A two-way (sex × genotype) ANOVA was then used to reveal the group differences in IA memory magnitudes, followed by Bonferroni’s *post hoc* tests if appropriate. However, two-way (empty vs. stranger 1 × group and stranger 1 vs. stranger 2 × group) ANOVAs were used to reveal group differences in sociability and social novelty preference, followed by Bonferroni’s *post hoc* tests if appropriate. Another two-way (sex × genotype) ANOVA was used to reveal group differences in preference ratios followed by Bonferroni’s *post hoc* tests if appropriate. In addition, a two-way (sex × genotype) ANOVA was used to analyze group differences in total exploration time spent on this task. Likewise, two-way (sex × genotype) ANOVAs were exploited otherwise to assess sex and SEPT14 KO effects on object location memory, tail suspension immobility time, novelty-induced FR, observational fear conditioning magnitude, observational learning-induced CORT secretion, open field traveling distance, velocity, and time spent in arena center, dorsal, and ventral hippocampal DG stem cell proliferation and protein expression followed by Bonferroni’s *post hoc* tests if appropriate. All the level of statistical significance was set at *p* < 0.05.

## Results

### Generation of *Septin-14* Knockout Mice

**FIGURE 1:**
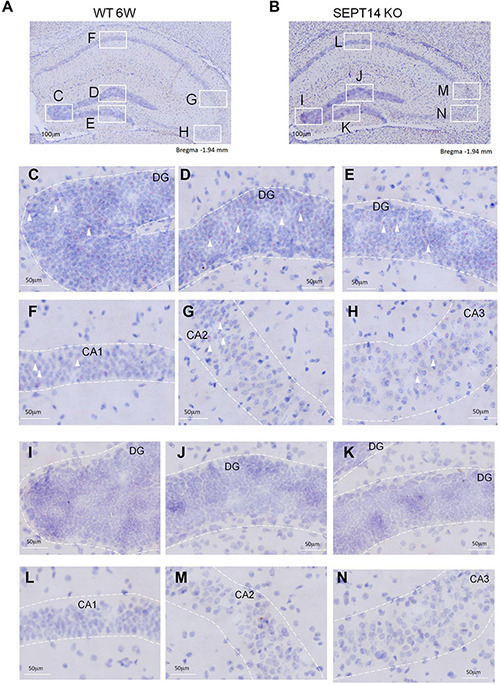
Distribution of *Septin-14* mRNA in the hippocampus. **(A,C–H)** BaseScope images of hippocampus sections from WT mice **(A)**. The outlined areas were enlarged to show regions of DG **(C–E)**, CA1 **(F)**, CA2 **(G)**, and CA3 **(H)** in panel **A**. Punctated red dots indicate the presence of *Septin-14* mRNAs (white arrows). **(B,I–N)** BaseScope images of hippocampus sections from SEPT14 KO mice **(B)**. The outlined areas were enlarged to show regions of DG **(I–K)**, CA1 **(L)**, CA2 **(M)**, and CA3 **(N)** in panel **(B)**.

We first generated SEPT14-deficient mice using the CRISPR/Cas9 KO strategy (Supplementary Figure 1A). Deletion of SEPT14 was verified using PCR (Supplementary Figure 1B). Given the expression of SEPT14 in the testes and brain (Shinoda et al., 2010), RT-PCR was used to examine the SEPT14 expression in these two tissues. We found SEPT14 transcript was abolished in the SEPT14 KO hippocampus, cerebellum (Supplementary Figure 1C), prefrontal cortex (data not shown), and testes (data not shown). A previous study has shown that SEPT14 is expressed in the cortical plate of the developing cerebral cortex (Shinoda et al., 2010). Analysis of the raw data from large-scale single-cell RNA-sequencing work (Yao et al., 2021) indicates that the anterolateral motor cortex and hippocampal region express low levels of *Sept14*. Given these findings, we used BaseScope *in situ* hybridization to examine the regional expression pattern of *Sept14* mRNA in the adult mouse brain. *Sept14* mRNA was detected in the DG, hippocampal CA1–CA3 (Figures 1A,C–H), and cerebral cortex (Supplementary Figures 2A,C–E) in WT mice. In contrast, no signal for *Sept14* mRNA was detected in the hippocampus (Figures 1B,I–N) and cerebral cortex (Supplementary Figures 2B,F–H) in SEPT14 KO mice, supporting the specificity of the *Sept14* mRNA probe used for BaseScope. The body weight and brain weight were not significantly different between female WT and SEPT14 KO mice. The brain weight was not different between WT and KO mice in males. There were no gross abnormalities in the brain in the KO mice. The body weight was slightly increased while the brain/body weight ratio was slightly decreased in the 8-week old male SEPT14 KO mice (Supplementary Figure 3). Some KO mice have been observed up to 32 weeks of age. There were no obvious health problems in these mice. Both SEPT14^–^/^–^ male and female mice were fertile (data not shown). It seems SEPT14 is not essential for development, fertility, or viability.

### Male, but Not Female, *Septin-14* Knockout Mice Had Elevated Inhibitory Avoidance Magnitude as Compared to Same-Sex Wild-Type Mice

**FIGURE 2:**
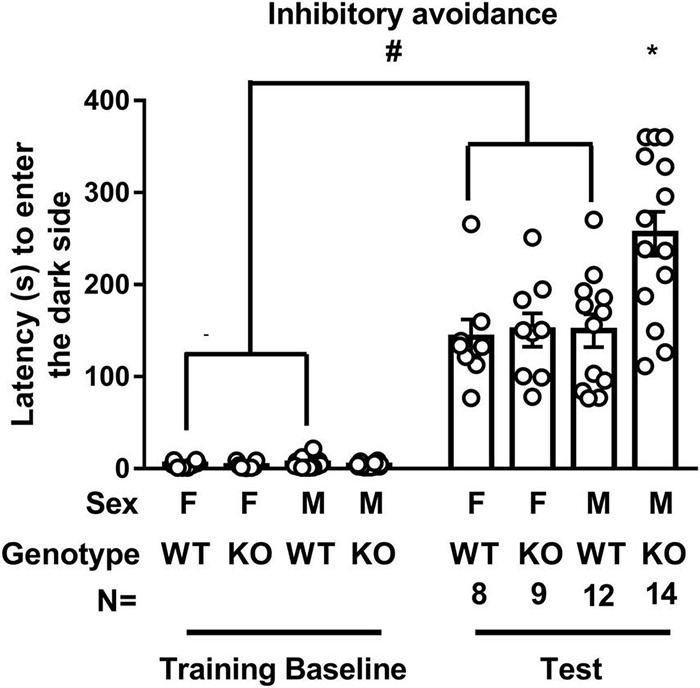
Inhibitory avoidance training and test latency to enter the dark compartment. WT mice’ latency to enter the dark compartment in the test was significantly longer than it was in the training baseline. SEPT14 KO did not seem to affect the latency to enter the dark compartment in female mice, while this SEPT14 KO enhanced the latency to enter the dark compartment in male mice. F, M, WT, and KO are short forms of female, male, wild type, and Septin-14 knockout, respectively. ^#^Significantly higher than training baseline (*p* < 0.05). *Significantly higher than the remaining three groups in the test (*p* < 0.05). The bar and error bar represent the group mean and standard error of mean (SEM), respectively.

Regardless of sex, WT mice’ latency to enter the dark compartment in the IA test was significantly longer than it was in the training baseline [*t*(38) = 10.87, *p* < 0.0001], suggesting the validity of our IA training protocol. While SEPT14 KO did not seem to affect the latency to enter the dark compartment in female mice, male SEPT14 KO mice displayed enhanced latency to enter the dark compartment [*F*(1,39) = 4.983, *p* = 0.0314], suggesting that SEPT14 KO is associated with elevated aversion-related memory exclusively in male mice (Figure 2).

### Male, but Not Female, *Septin-14* Knockout Mice Had Higher Hippocampal mGluR5 Expressions as Compared With the Same-Sex Wild-Type Mice

**FIGURE 3:**
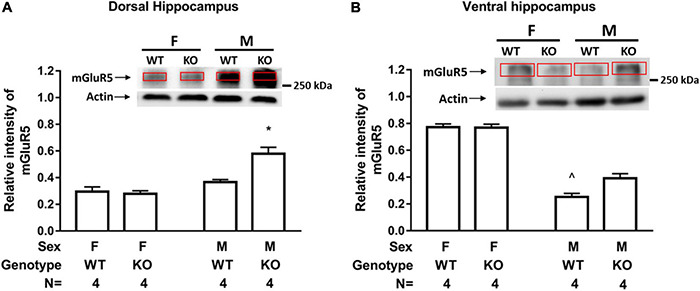
Hippocampal mGluR5 level in two sexes of WT and SEPT14 KO mice. **(A)** Male SEPT14 KO mice demonstrated elevated mGluR5 levels in dorsal hippocampal samples. *Significantly greater than the remaining three groups (*p* < 0.05). **(B)** Male SEPT14 KO mice demonstrated elevated mGluR5 levels in ventral hippocampal samples. ^∧^Significantly lower than the remaining three groups (*p* < 0.05). The bar and error bar represent group mean and SEM, respectively.

Male and female WT and SEPT14 KO mice exhibited comparable GluA1 and GluA2 levels in tissues containing dorsal and ventral hippocampus (Supplementary Figure 4). A two-way (sex × genotype) ANOVA revealed that there was an interaction effect of sex and genotype on dorsal hippocampal mGluR5 level [*F*(1,12) = 12.17, *p* = 0.0045]. *Post hoc* tests further revealed that male SEPT14 KO mice exhibited greater mGluR5 levels as compared to same-sex WT mice in tissues containing the dorsal hippocampus (Figure 3A). Likewise, an interaction effect of sex and genotype was noticed on the ventral hippocampal mGluR5 level [*F*(1,12) = 6.681, *p* = 0.0239]. *Post hoc* tests further indicated that male SEPT14 KO mice had higher mGluR5 levels in the ventral hippocampus as compared to same-sex WT mice (Figure 3B).

### Both Male and Female *Septin-14* Knockout Mice Had Dampened Observational Fear Conditioning Magnitude (Conditioned Vanilla Odor-Induced Freezing Response)

**FIGURE 4:**
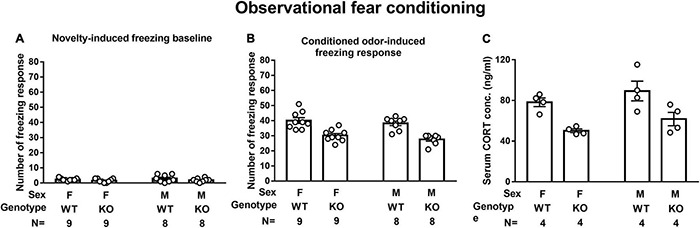
Novelty- and observational conditioning-induced freezing response (FR) and observational fear conditioning-induced corticosterone (CORT) secretion. **(A)** Regardless of sex and genotype, mice had indistinctive novelty-induced freezing baselines. **(B)** SEPT14 KO mice had dampened conditioned vanilla odor-induced FR as compared to WT mice [*F*(1,30) = 45.48, *p* < 0.0001]. **(C)** SEPT14 KO mice had dampened observational conditioning-stimulated CORT secretion as compared with the WT ones [*F*(1,12) = 19.54, *p* = 0.0008]. The bar and error bar represent group mean and SEM, respectively.

A two-way (sex × genotype) ANOVA revealed that neither sex nor genotype affected novelty-induced freezing baseline in a 10-min test. Male and female WT mice demonstrated comparable odor-induced FRs (Figure 4A). However, a main effect of genotype was noted on odor-induced FR [*F*(1,30) = 45.48, *p* < 0.0001], suggesting that male SEPT14 KO mice displayed lower vanilla odor-induced FRs as compared to their same-sex WT controls regardless of their sexes (Figure 4B). While male and female WT mice had comparable serum CORT levels immediately after the first round of observational learning, male and female SEPT14 KO mice had lower serum CORT levels as compared to their same-sex WT mice [*F*(1,12) = 19.54, *p* = 0.0008] (Figure 4C). These results, taken together, suggest that SEPT14 KO appears to dampen observation-related stress-induced CORT secretion and observational fear learning and memory magnitude in both male and female mice.

### Male and Female *Septin-14* Knockout Mice Had Enhanced Neural Stem Cell Proliferation in Ventral, but Not Dorsal, Hippocampal Dentate Gyrus

**FIGURE 5:**
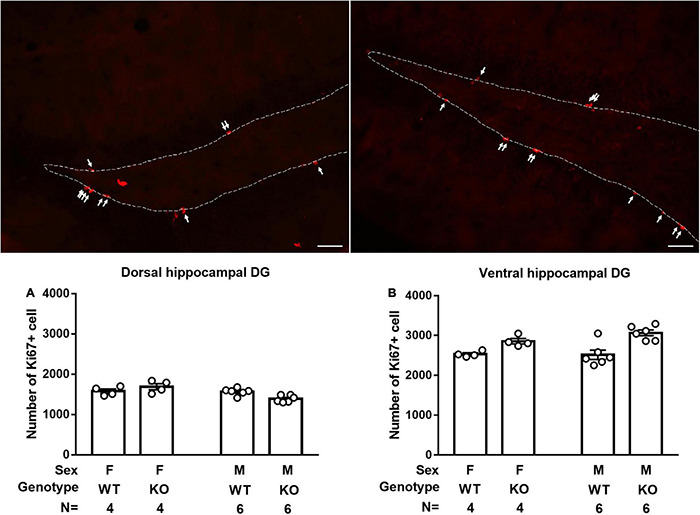
Dorsal and ventral hippocampal cell proliferation in WT and SEPT14 KO mice. Microphotograph representatives of dorsal (top left) and ventral (top right) DG with white arrows pointing to Ki67-positive cells. **(A)** Male and female SEPT14 KO mice had comparable numbers of newly proliferated cells in dorsal hippocampal DG. **(B)** SEPT14 KO mice had greater newly proliferated cells as compared with their same-sex WT mice in ventral hippocampal DG [*F*(1,12) = 21.43, *p* = 0.0006]. The bar and error bar represent group mean and SEM, respectively.

Male and female WT mice had indistinctive numbers of Ki67-positive cells in the dorsal and ventral hippocampal DG (Figure 5A). SEPT14 KO and WT mice had an indistinctive number of newly proliferated cells in the dorsal hippocampal DG in male and female mice (Figure 5A). In contrast, SEPT14 KO mice had greater newly proliferated cells than that same-sex WT mice in ventral hippocampal DG, regardless of sex [*F*(1,16) = 23.17, *p* = 0.0002] (Figure 5B).

### Both Male and Female *Septin-14* Knockout Mice Demonstrated Heightened Anxiety Levels in Lit Open Field Free Navigation Task

**FIGURE 6:**
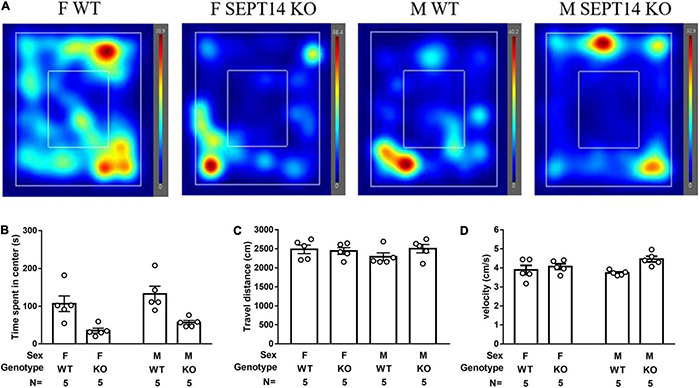
Stress-provoked anxiety level and locomotion in open field free navigation task. **(A)** Navigation heating map representatives were listed. **(B)** SEPT14 KO mice exhibited less time spent navigating in the center as compared to their same-sex WT ones [*F*(1,12) = 21.43, *p* = 0.0006]. Four groups of mice demonstrated comparable **(C)** traveling distance and **(D)** velocity in this navigation task. The bar and error bar represent group mean and SEM, respectively.

A two-way (sex × genotype) ANOVA revealed that the two sexes of SEPT14 KO mice seemed to exhibit significantly less time spent navigating in the lighted center as compared to their same-sex WT ones [*F*(1,12) = 21.43, *p* = 0.0006] (Figures 6A,B). Regardless of genotypes (WT and SEPT14 KO), both male and female mice demonstrated comparable navigation distance (Figure 6C) and velocity (Figure 6D) in the stress-related navigation task.

### *Septin-14* Knockout Did Not Seem to Affect Object Location Performance or Tail Suspension Immobility Time

**FIGURE 7:**
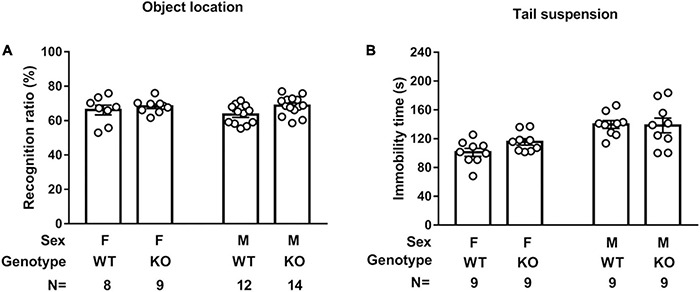
SEPT14 KO and object location memory and tail suspension immobility time. **(A)** Four groups of mice showed indistinctive recognition ratios in the object location test session. **(B)** Male mice exhibited greater immobility time than female mice in the tail suspension task. SEPT14 KO did not affect such immobility time in either sex. Bar and error bar represent group mean and SEM, respectively.

A two-way ANOVA revealed that mice showed indistinctive recognition ratios in test sessions regardless of their sexes and genotypes, suggesting that sex or SEPT14 KO did not affect spatial location memory (Figure 7A). A two-way (sex × genotype) ANOVA revealed that male mice seemed to exhibit greater immobility time than female mice in the tail suspension task [*F*(1,32) = 20.36, *p* < 0.0001] (Figure 7B). Nonetheless, SEPT14 KO did not affect such immobility time in either sex.

### *Septin-14* Knockout Did Not Affect Sociability or Social Novelty Preference

**FIGURE 8:**
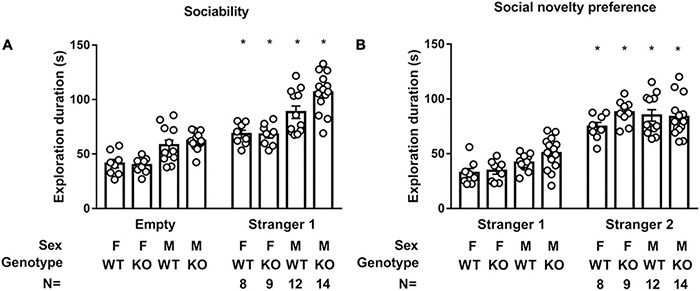
SEPT14 KO and sociability and social novelty preference. **(A)** While all four groups of mice spent higher exploration duration on stranger 1 than on empty cage [*F*(1,78) = 112.9, *p* < 0.0001], male mice spent higher total exploration duration toward stranger 1 and empty cage as compared to female mice [*F*(1,39) = 45.31, *p* < 0.0001]. *Significantly higher than their exploration duration with an empty cage (*p* < 0.05). **(B)** All four groups of mice spent higher exploration duration on stranger 2 than previously met stranger 1 [*F*(1,78) = 198.7, *p* < 0.0001]. *Significantly greater than their exploration time with stranger 1 cage (*p* < 0.05). Male mice spent higher total exploration duration to stranger 1 and 2 cages as compared to female mice [*F*(1,39) = 4.798, *p* = 0.0345]. The bar and error bar represent group mean and SEM, respectively.

Regardless of sex and genotype, all four groups of mice spent a higher exploration duration on stranger 1 than on an empty cage [*F*(1,78) = 112.9, *p* < 0.0001], suggesting neither sex nor SEPT14 KO affected mice’ sociability (Figure 8A). Interestingly, male mice spent higher total exploration duration in empty and stranger 1 cages as compared to female mice [*F*(1,39) = 45.31, *p* < 0.0001]. The latter results imply that female mice seem to exhibit stronger neophobia. Likewise, a two-way (group × genotype) ANOVA revealed that all mice spent higher exploration duration on stranger 2 than on previously met stranger 1 [*F*(1,78) = 198.7, *p* < 0.0001] (Figure 8B). Interestingly, male mice spent a higher total exploration duration toward stranger 1 and 2 cages as compared to female mice [*F*(1,39) = 4.798, *p* = 0.0345], suggesting females’ stronger neophobia. The implications of these findings, taken together, suggest that sex and SEPT14 KO do not seem to affect sociability or social novelty preference, while female mice appear to have stronger neophobia.

## Discussion

Contrasting WT and SEPT14 KO mice in their performances in an observational fear conditioning task, we found that male and female WT mice had comparable novelty-provoked naïve fear responses and observational fear conditioning magnitudes. Male and female SEPT14 KO mice displayed comparable novelty-provoked naïve fear responses, while male and female SEPT14 KO mice had lower observational fear conditioning magnitudes as compared to their same-sex WT mice. Since observational fear conditioning-provoked CORT secretion seems to be a prerequisite for the reliable formation of such observational learning (Liao et al., 2021b), we assessed observational learning-provoked CORT secretion in WT and SEPT14 KO mice following the conclusion of the first training session. Interestingly, male and female SEPT14 KO mice, in fact, had lower observational learning trial-stimulated CORT secretion as compared to their same-sex WT mice. These findings imply that SEPT14 KO may impede observational fear conditioning magnitude by attenuating animals’ CORT secretion-related attention to demonstrators’ experiences and/or CORT secretion-related memory consolidation (Lesuis et al., 2018; de Tommaso et al., 2019; Merino et al., 2020). Accordingly, the causative relationship between Septin-14 intactness and attention-coordinating prefrontal cortex (Wulaer et al., 2020) and memory consolidation-related hippocampus (Keller et al., 2015) throughout development deserves further study.

The hippocampal DG is one of the primary brain regions harboring neural stem cells. Active proliferation of these neural stem cells in dorsal hippocampal DG seems to play a minor role in adult anxiety-like behaviors (Kozareva et al., 2019). In contrast, active stem cell proliferation in ventral hippocampal DG is found to correlate positively with the magnitudes of anxiety-like behavior (Morley-Fletcher et al., 2011; Zhao et al., 2018), suggesting a potential, anxiety-modulating role of ventral hippocampal stem cell proliferation. Nonetheless, one line of evidence has shown that dampening ventral hippocampal DG stem cell proliferation does not seem to affect mice’ depressive-like behaviors (Mateus-Pinheiro et al., 2017). Moreover, ventral hippocampal DG cell proliferation does not seem to participate in stress-provoked anxiety-like behaviors in a rat model (Mori et al., 2020). Furthermore, ventral hippocampal DG cell proliferation is loosely associated with stress-produced long-lasting anxiety-like phenotypes in another rat model (Schoenfeld et al., 2019). As compared to the same-sex WT mice, we found that both sexes of SEPT14 KO mice exhibited increases in ventral hippocampal cell proliferation and decreases in stress-produced center exploration time spent on open field tasks in this study. It is important to note that SEPT14 deletion did not seem to affect mice’ locomotion and moving velocity in this regard. Thus, SEPT14 KO mice’s anxiety-prone behavioral phenotype may not be attributed to their locomotor ability or exploratory motivation changes. Our findings, taken together, appear to support the scenario (Schoenfeld et al., 2019; Mori et al., 2020) that ventral hippocampal stem cell proliferation may play, at best, a minor role in modulating anxiety-like behavioral phenotypes. Stress-induced immobility duration has been regarded as rodents’ convenient depression-like behavioral model (Liao et al., 2021c). We found that SEPT14 KO did not seem to affect immobility duration using such rodents’ depression model.

We noticed that sex and SEPT14 KO did not seem to affect mice’ sociability and social novelty preference. Interestingly, male mice were found to spend greater exploration duration toward empty and stranger 1 cages in combination and stranger 1 and 2 cages in combination than female mice. Regardless of SEPT14 KO, female mice seemed to exhibit a stronger phobic phenotype to the introduction of a novel conspecific. Compared with male mice, it has been found that female mice may demonstrate stronger synaptic and stress-modulated behavioral plasticity primarily due to their differences in gonadal hormones (Chen et al., 2014). Since we did not identify female mice’ estrous cycles or their sex hormone levels prior to any of the behavioral tasks, an extended study warrants to be done to confirm such sex differences in this neophobia to the presence of a new conspecific and other sexually dimorphic effects observed in this study.

To date, glutamate AMPA and mGluR5 receptor expression are thought to be positively associated with the formation of IA memory. For instance, downregulation of hippocampal glutamate GluA1 and GluA2 subunits of the AMPA receptor has been demonstrated to account for drug- and intoxicant-induced IA disruption in rat models (Chen et al., 2001; Beheshti et al., 2020). Moreover, dorsal hippocampal glutamate AMPA receptor upregulation appears to be a permissive and necessary neurochemical plasticity participating in forming an IA memory (Cammarota et al., 1995). Furthermore, mGluR5 knockout mice have been demonstrated to exhibit aberrant performance in the formation and retention of aversive emotion-related memories and impaired hippocampal glutamatergic circuits (Xu et al., 2009). In this study, we noticed that male and female mice, regardless of their genotype (WT vs. SEPT14 KO), demonstrated comparable GluA1 and GluA2 levels in the dorsal and ventral hippocampus. Interestingly, SEPT14 KO seemed to exert sexually dimorphic effects on inhibitory memory and dorsal and ventral hippocampal mGluR5 expression. Paradoxically, male, but not female, SEPT14 KO mice displayed elevated IA memory magnitude and dorsal/ventral hippocampal mGluR5 levels. In parallel with those findings that hippocampal mGluR5 is involved in IA memory, in this study, we reported that male SEPT14 KO mice exhibited elevated IA memory magnitude and upregulated hippocampal mGluR5 expression. In addition to our findings that there may be sexually dimorphic roles of Septin-14 in dorsal hippocampal mGluR5 expression, a few lines of evidence point out that estradiol administration may affect mGluR5 signaling-mediating neuronal morphological plasticity and behavior in rat models (Peterson et al., 2015; Martinez et al., 2016). We suspect that the sexually dimorphic effects of SEPT14 KO on mGluR5 expression may be due to adult mice’ sex hormone differences. Well-designed studies should be conducted to assess this hypothesis. Likewise, it is worthwhile to assess whether deletion of the *SEPT14* gene may render mGluR5 upregulation as early as early life and/or adolescence of development (Yu et al., 2002). In addition to sex hormones, sex-specific involvement of synaptic protein kinases, transcription factors, and thus gene expressions in synaptic plasticity have been noted to be associated with sexually dimorphic behavioral and physiological phenotypes (Mizuno and Giese, 2010). Septin-associated protein kinases have been known to play a paramount role in orchestrating cell proliferation processes and cellular morphogenesis (Perez et al., 2016). Likewise, two septin family members have been demonstrated to interact with MEK/ERK, kinases involved in gene expression-regulating signaling pathways (Zhang et al., 2016). We, thus, conjecture that SEPT14 may affect mGluR5 expression by interacting with protein kinases. In an attempt to further understand the association between SEPT14 KO and mGluR5 upregulation, it is mandatory to assess the plausible impact of SEPT14 deletion on synaptic kinases and downstream transcription factors.

## Conclusion

These results, taken together, prompt us to conclude that deletion of the *Septin-14* gene may render elevated ventral hippocampal DG cell proliferation and stress-provoked anxiety while dampening observational fear conditioning magnitudes in both sexes of adult animals. In addition, mice with *Septin-14* gene deletion may exhibit elevated aversive emotion-supported memory magnitude and dorsal/ventral hippocampal mGluR5 expression exclusively in male, but not female, animals.

## Data Availability Statement

The raw data supporting the conclusions of this article will be made available by the authors, without undue reservation.

## Ethics Statement

All procedures were approved by the Local Animal Care Committee at National Cheng Kung University College of Medicine (NCKUCM No. 107172).

## Author Contributions

K-RC, H-YW, P-LK, and LY conceived and designed the experiments and wrote the manuscript. K-RC, H-YW, Y-HL, Y-HH, and L-HS conducted the experiments. Y-HL, P-LK, and LY analyzed the data. K-RC, P-LK, and LY gave final approval to the revised version to be published. All authors contributed to the article and approved the submitted version.

## Conflict of Interest

The authors declare that the research was conducted in the absence of any commercial or financial relationships that could be construed as a potential conflict of interest.

## Publisher’s Note

All claims expressed in this article are solely those of the authors and do not necessarily represent those of their affiliated organizations, or those of the publisher, the editors and the reviewers. Any product that may be evaluated in this article, or claim that may be made by its manufacturer, is not guaranteed or endorsed by the publisher.
