# Supplementary material for: Effects of Septin-14 Gene Deletion on Adult Cognitive/Emotional Behavior
Source: Front Mol Neurosci. 2022 Apr 29;15:880858. doi: 10.3389/fnmol.2022.880858 (PMC9100402; doi:10.3389/fnmol.2022.880858)
Supplement: Supplementary file 1 [file Data_Sheet_1.docx]

Supplementary Material


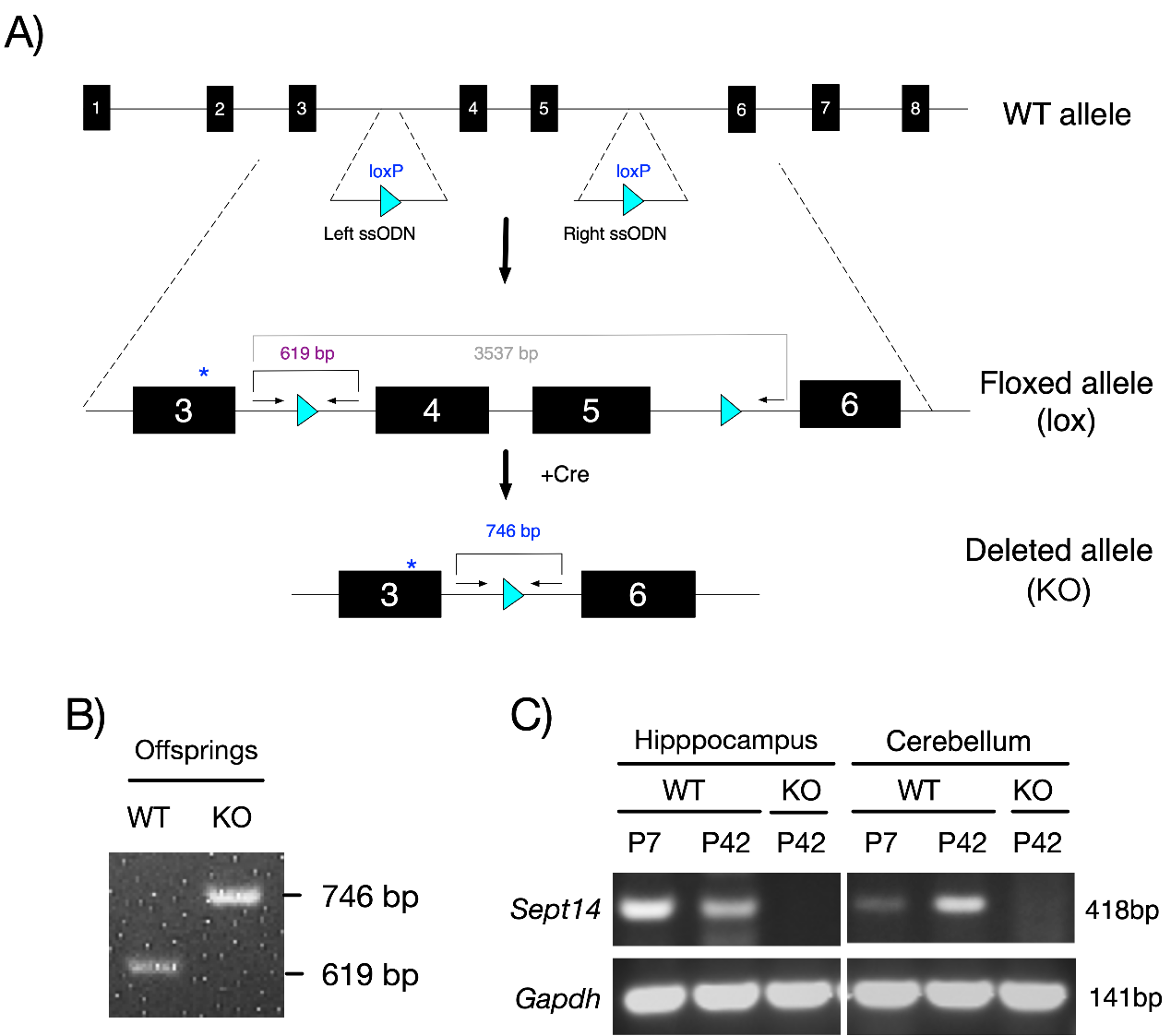


**Supplementary Figure 1.** Generation of SEPT14–deficient mice. (A) Scheme of the strategy for generating animals carrying the SEPT14 knockout allele. (B) The SEPT14 knockout allele was analyzed by PCR. (C) Endogenous SEPT14 mRNA levels in WT and SEPT14 knockout hippocampus and cerebellum were analyzed by RT-PCR.


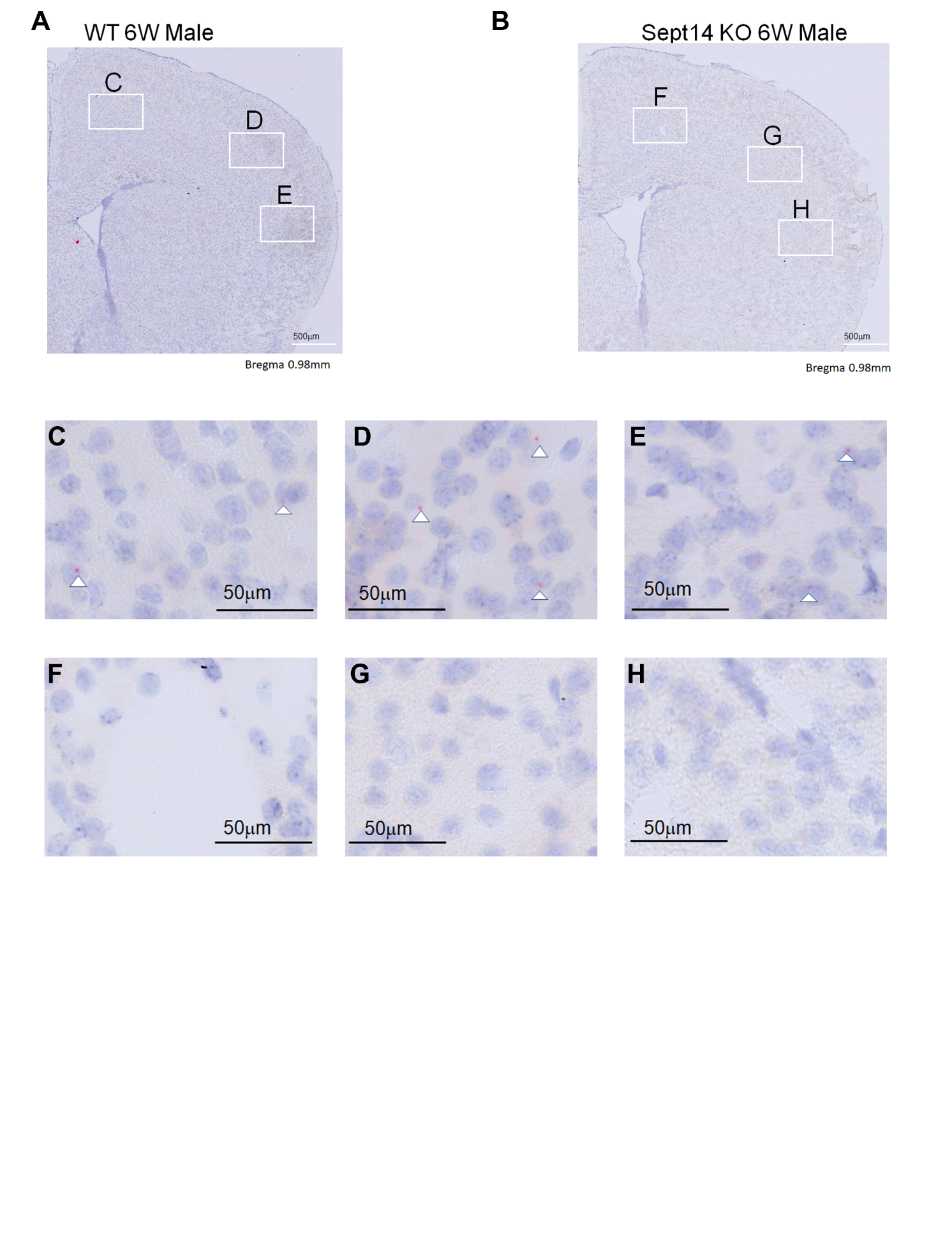


**Supplementary Figure 2.** **Distribution of *Septin 14* mRNA in the cerebral cortex.** (A, C-E) BaseScope images of hippocampus sections from WT mice (A). The outlined areas were enlarged to show regions of cerebral cortex (C-E) in panel A. Punctate red dots indicate the presence of *septin 14* mRNAs (white arrows). (B, F-H) BaseScope images of hippocampus sections from SEPT14 KO mice (B). The outlined areas were enlarged to show regions of cerebral cortex (F-H) in panel B.


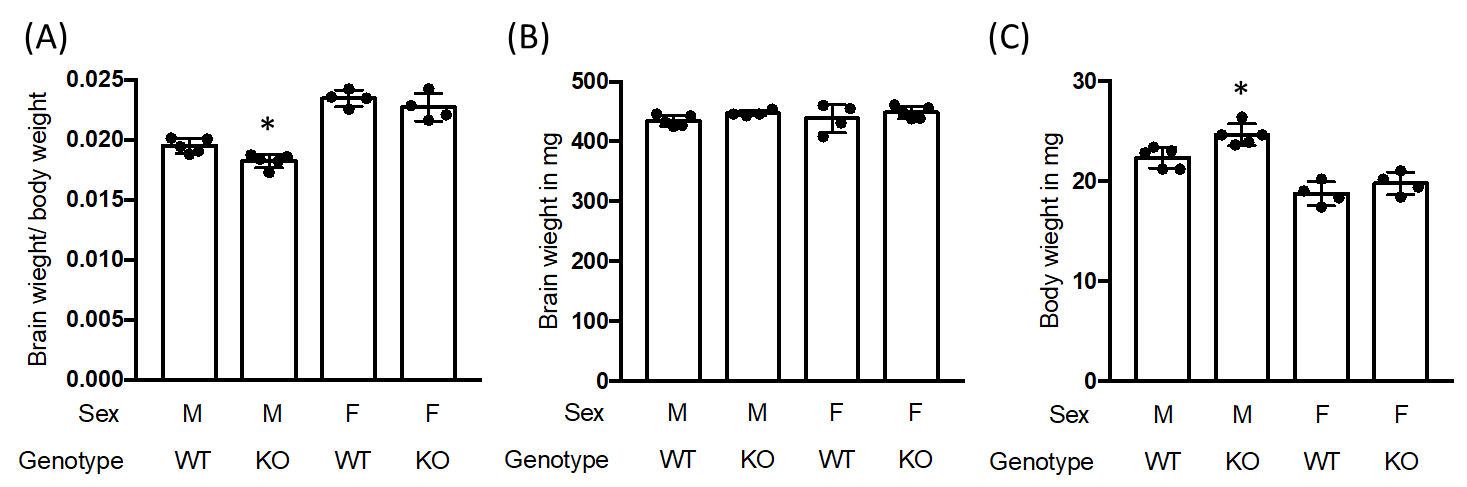


**Supplementary Figure 3.** Brain/body weight ratio, brain weight and body weight were measured in WT and SEPT14 KO mice. (A-C) Brain weight and body weight (WT; male n = 5, SEPT14 KO; male n = 5, WT; female n = 4, SEPT14 KO; female n = 4,) of the mice were measured at 8-weeks-of-age. (A) SEPT14 KO males brain weight / body weight less than age-matched WT males. (B and C) SEPT14 KO did not affect brain weight (B). SEPT14 KO males weighed more than age-matched WT males (C). Overall group were carried out using two-way ANOVA followed by Bonferroni's post hoc tests. *p < 0.05 versus WT of the same sex.


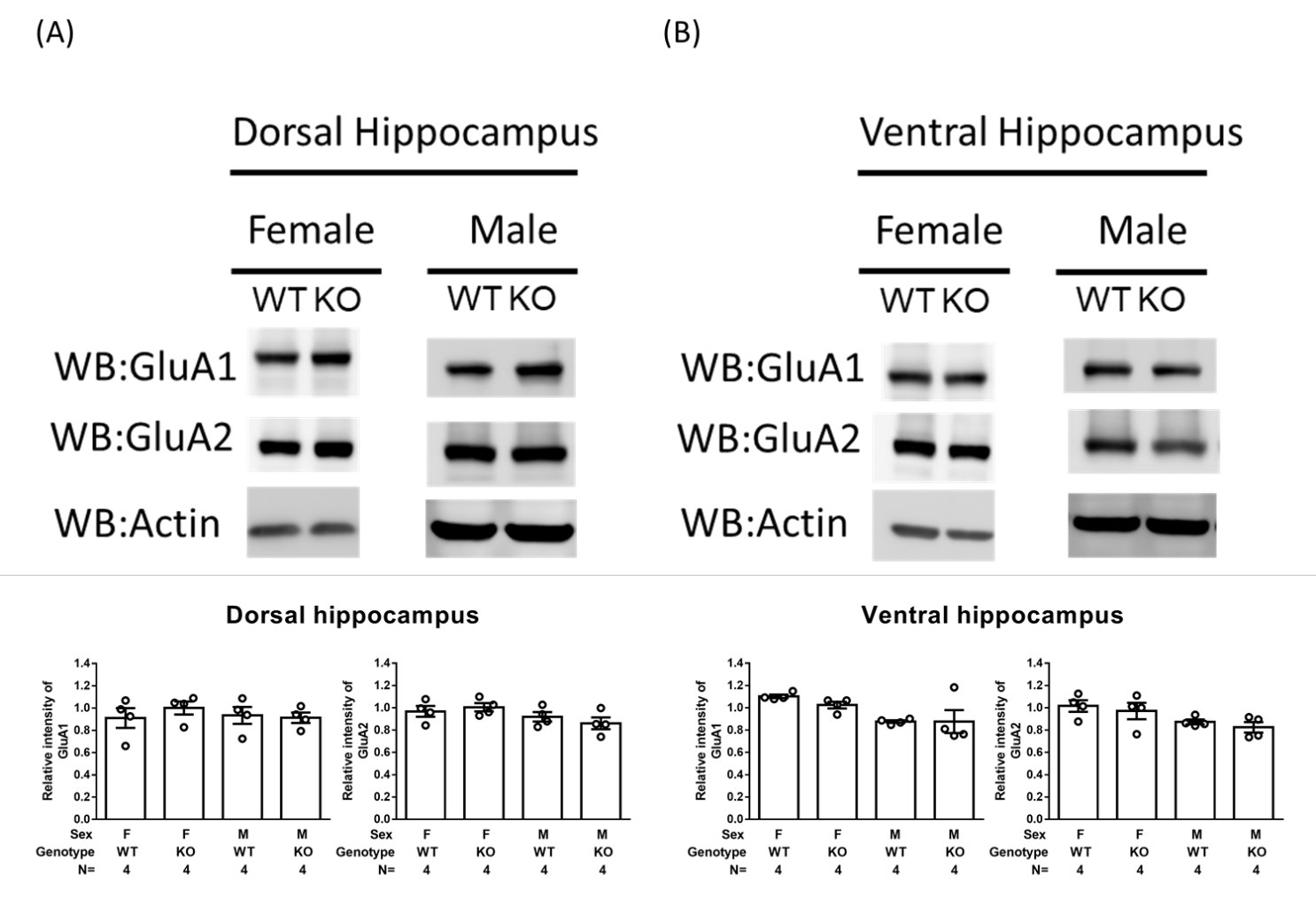


**Supplementary Figure 4.** Hippocampal GluA1 and GluA2 levels in two sexes of WT and SEPT14 KO mice. (A and B) Representative western blots for total GluA1 and GluA2 and their respective actin in the dorsal (A) and ventral (B) hippocampus of WT and SEPT14 KO mice. (A) Regardless of sex and genotype, a two-way (sex x genotype) ANOVA revealed that 4 groups of mice demonstrated comparable GluA1 and GluA2 levels in dorsal hippocampal tissues. (B)Likewise, another two-way (sex x genotype) ANOVA revealed that 4 groups of mice demonstrated comparable GluA1 and GluA2 levels in ventral hippocampal tissues.
